# From KIDSCREEN-10 to CHU9D: creating a unique mapping algorithm for application in economic evaluation

**DOI:** 10.1186/s12955-014-0134-z

**Published:** 2014-08-29

**Authors:** Gang Chen, Katherine Stevens, Donna Rowen, Julie Ratcliffe

**Affiliations:** Flinders Health Economics Group, School of Medicine, Flinders University, Adelaide, Australia; Health Economics and Decision Science, School of Health and Related Research, University of Sheffield, Sheffield, UK; A Block, Level 1, Repatriation General Hospital, School of Medicine, Flinders University, Daws Road, Daw Park, SA 5041 Australia

**Keywords:** Health-related quality of life, CHU9D, KIDSCREEN, Mapping, Utility, Adolescent

## Abstract

**Background:**

The KIDSCREEN-10 index and the Child Health Utility 9D (CHU9D) are two recently developed generic instruments for the measurement of health-related quality of life in children and adolescents. Whilst the CHU9D is a preference based instrument developed specifically for application in cost-utility analyses, the KIDSCREEN-10 is not currently suitable for application in this context. This paper provides an algorithm for mapping the KIDSCREEN-10 index onto the CHU9D utility scores.

**Methods:**

A sample of 590 Australian adolescents (aged 11–17) completed both the KIDSCREEN-10 and the CHU9D. Several econometric models were estimated, including ordinary least squares estimator, censored least absolute deviations estimator, robust MM-estimator and generalised linear model, using a range of explanatory variables with KIDSCREEN-10 items scores as key predictors. The predictive performance of each model was judged using mean absolute error (MAE) and root mean squared error (RMSE).

**Results:**

The MM-estimator with stepwise-selected KIDSCREEN-10 items scores as explanatory variables had the best predictive accuracy using MAE, whilst the equivalent ordinary least squares model had the best predictive accuracy using RMSE.

**Conclusions:**

The preferred mapping algorithm (i.e. the MM-estimate with stepwise selected KIDSCREEN-10 item scores as the predictors) can be used to predict CHU9D utility from KIDSCREEN-10 index with a high degree of accuracy. The algorithm may be usefully applied within cost-utility analyses to generate cost per quality adjusted life year estimates where KIDSCREEN-10 data only are available.

## Background

Health-related quality of life (HRQoL) is a multidimensional construct that measures the impact of health or disease on physical and psychosocial functioning [1,2]. The measurement and valuation of HRQoL is a major issue for health services research and has become an essential component for assessing the cost-effectiveness of treatments and interventions in public health and clinical medicine research internationally [3]. HRQoL instruments can be categorised into two groups: health profile measures providing simple summative index summary scores for individual dimensions (items) and/or overall health, and preference based instruments/multi-attribute utility instruments containing preference weights for individual dimensions relative to each other and a preference weighted summary score for each health state defined by the instrument. Multi-attribute utility instruments can be used to generate quality adjusted life years (QALYs) for use in cost-utility analyses. QALYs are the preferred outcome measure for many regulatory bodies including the National Institute for Health and Clinical Excellence in the UK and the Pharmaceutical Benefits Advisory Committee in Australia [3,4].

The majority of HRQoL instruments developed specifically for children and adolescent populations are not suitable for application within the framework of cost-utility analysis because they are non-preference based. One of the most prevalent non-preference based instruments, widely used in both public health and clinical medicine disciplines across countries, is the KIDSCREEN [5-8]. The KIDSCREEN has a simple summative scoring system in which equal weights are attached to different dimensions of HRQoL. However, a valid instrument that can be used to generate QALYs in cost-utility analyses needs to have the ability to ‘measure’ health status and also the ability to ‘value’ health status by incorporating preferences relating to the relative desirability of the dimensions and severity levels of each of the dimensions included in the instrument.

Mapping or cross walking techniques may be applied to link profile instruments and preference based instruments together thereby enabling non-preference based HRQoL instrument results to be utilised within the framework of cost-utility analyses [4,9]. A comprehensive review by Brazier and colleagues [9] identified 30 mapping studies in the literature. All of these studies had been conducted using instruments designed for measuring HRQoL in adults, and had been applied exclusively in adult populations. To date, only one previous study has conducted a mapping exercise exclusively in a paediatric population. Furber and colleagues mapped the Strengths and Difficulties Questionnaire responses into Child Health Utility 9D (CHU9D) utilities [10].

The main objective of this study was to develop an algorithm for generating CHU9D utility scores from KIDSCREEN-10 index summary scores, facilitating cost-utility analyses within studies where health outcomes are assessed only by the KIDSCREEN-10 index.

## Methods

### Study design

An online survey was developed for administration to a community based sample of adolescents living in Australia, aged 11–17 years. Following parent and adolescent consent, adolescents were invited to complete a survey which included the CHU9D and KIDSCREEN-10 instruments, socio-demographic variables (gender, age and socio-economic status as measured by the Family Affluence Scale) [11], a five-scale self-reported general health question (measured as excellent, very good, good, fair and poor), and whether they had a long standing disability, illness or medical condition. This study was approved by the Social and Behavioural Research Ethics Committee, Flinders University (project number 4701).

### Instruments

The KIDSCREEN-10 is a generic non-preference based measure of well-being and HRQoL developed internationally for children and adolescents aged 8 to 18 years old [5]. It is a short version of the KIDSCREEN-52 and KIDSCREEN-27 instruments and has demonstrated criterion validity, convergent validity and known groups validity [12,13]. The KIDSCREEN-10 contains 10 items: fit and well (KS_I1), energy (KS_I2), sad (KS_I3), lonely (KS_I4), had enough time for yourself (KS_I5), been able to do the things that you want to do in your free time (KS_I6), parent(s) treated you fairly (KS_I7), had fun with friends (KS_I8), got on well at school (KS_I9) and been able to pay attention (KS_I10), each with a 5 point response scale [13]. The calculation of KIDSCREEN-10 index involve three steps: firstly, a raw overall score is summed by adding each item score with equal weight; secondly, the sum scores are converted to a score by assigning Rasch person parameters to each possible sum score; and lastly, the person parameters are transformed into values with a mean of approximately 50 and standard deviation approximately 10 [12]. A higher score is indicative of a better HRQoL. Both self-reported and parent proxy versions are available for KIDSCREEN instruments. The self-reported version was adopted in this study.

The CHU9D is a newly developed generic preference based measure of HRQoL that was designed specifically for application with young people [14]. Whilst it was originally developed for use with younger children aged 7 to 11 years, recent studies have also demonstrated the practicality and validity of using CHU9D in older adolescent populations aged 11–17 years [15-17]. The CHU9D consists of 9 dimensions: worried, sad, pain, tired, annoyed, schoolwork/homework, sleep, daily routine, ability to join in activities, with 5 different levels representing increasing levels of severity within each dimension. The original health state valuation algorithm for CHU9D was generated from the application of the standard gamble method within the UK adult general population [18]. In this study, since Australian adolescent data is used, we applied a recently developed Australian adolescent specific scoring algorithm for the CHU9D instrument based upon the best-worst scaling method and anchored on the 1–0 full-health to dead scale using the UK standard gamble results [19]. The CHU9D utilities range between 0.33 and 1. The strength of overlap between the KIDSCREEN-10 and the CHU9D has been reported in detail elsewhere [17]. Briefly Stevens and Ratcliffe found a moderate degree of significant correlation between CHU9D utility scores and the KIDSCREEN-10 index (r = 0.61), with some differences in the coverage of the items for the respective descriptive systems. The KIDSCREEN-10 is broader in scope than the CHU9D which focuses on a narrower definition of HRQoL.

### Statistical analysis

To develop the mapping algorithm from the KIDSCREEN-10 index to CHU9D utility scores, a dataset containing responses to both instruments from the same individual is used to estimate the mapping algorithm that can then be applied to other studies. In this study two groups of models were considered. In the first group the CHU9D utility score was regressed upon the KIDSCREEN-10 index, and also a higher order of the KIDSCREEN-10 index if the relationship between the two instruments was found to be non-linear. In the second group the CHU9D utility score was regressed upon the individual KIDSCREEN-10 item raw response scores. In the event that not all KIDSCREEN-10 items coefficients were statistically significant, the stepwise regression with forward selection technique (with significance levels for entrance of 0.05) was used to choose the “best” combination of predictors from the 10 items [20]. In the mapping literature, Model 2 is the most widely used additive model [9]. In addition to individual item and overall summary scores several previous mapping studies have also included socio-demographic characteristics, in particular age and gender, to improve predictive performance [9]. The significance (or otherwise) of including age and gender was also considered here. To summarise, the following two models were considered.

$$ CHU9D=\alpha +{\beta}_1\cdot KS+{\beta}_2+K{S}^2+{\delta}_1\cdot A\mathit{\mathsf{g}}e+{\delta}_2\cdot Gender $$ (Model 1)

$$ CHU9D=\alpha +{\displaystyle {\sum}_{j=1}^k{\gamma}_j}\cdot KS\_ Ij\_ sw+{\delta}_1\cdot A\mathit{\mathsf{g}}e+{\delta}_2\cdot Gender $$ (Model 2)

where CHU9D is the CHU9D utility score, KS is the KIDSCREEN-10 index, KS^2^ is the KIDSCREEN-10 index squared, KS_Ij_sw are the selected KIDSCREEN-10 items based upon statistical significances using the stepwise regression technique, k is the number of selected KIDSCREEN-10 items. The significance level is set to be 5% in this study.

**Figure 1:**
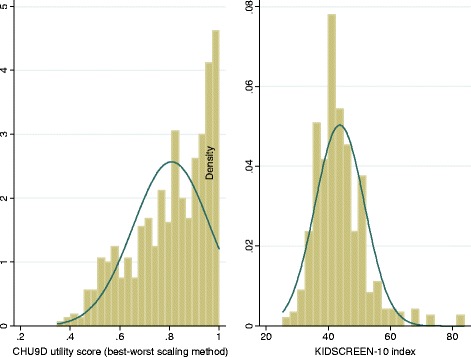
**Distribution of CHU9D utility scores and KIDSCREEN-10 index.**

Several econometric techniques have been adopted in previous studies to estimate mapping models, of which the ordinary least squares estimator has been the most widely adopted [9,21]. The majority of mapping models in the literature have mapped to EQ-5D, and as a result models are used that are appropriate for the distribution of EQ-5D responses which is typically bi-modal or tri-modal with a large proportion of responses at 1 (see Longworth and Rowen [22] for an overview). Figure 1 indicates that for this sample CHU9D responses are left-skewed with a large number of responses at 1. Appropriate estimators include: the Tobit estimator which takes into account bounding issues (e.g. for some multi-attribute utility instruments a high proportion of respondents report full health with a utility of 1), the censored least absolute deviations estimator which further relaxes the distributional assumption of the error term (i.e. not necessarily requiring the error term to be normal and homoscedastic as assumed by Tobit) [23,24], and the generalised linear model which allows for the non-normal distribution of dependent variables (e.g. left/negatively skewed utility scores) [25].

The ordinary least squares estimator is sensitive to potential outliers as it is based on the minimisation of the variance of the residuals. The censored least absolute deviations estimator mentioned above is a special case of robust regressions that does not suffer from this sensitivity and is therefore considered to be more suitable in this context. In this study we propose to include another effective robust estimator, the MM-estimator [26], that has been shown to have both a high breakdown point (i.e. the percentage of incorrect observations an estimator can handle before giving an incorrect result) and a high efficiency [27,28], but has not yet been utilised in mapping exercises. Heteroskedasticity robust standard errors are reported for inference.

Previous studies have indicated that the censored least absolute deviations estimator outperforms the Tobit estimator in relation to goodness-of-fit criteria (e.g. mean prediction error) (see for example Sullivan and Ghushchyan [29]). However since no other definitive evidence is available regarding the superiority of a particular estimator, we chose to utilise four estimators (ordinary least squares, censored least absolute deviations, MM and generalised linear model) in this study. Among different combinations of *family* and *link* function for the generalised linear model, the binomial family with logit link was chosen as the most appropriate since it showed the best performance of predicting the mean utility close to the observed. Regression analyses were estimated in Stata version 12.1 (StataCorp LP, College Station, Texas, USA).

Goodness-of-fit was examined using mean absolute error (MAE) and root mean square error (RMSE) – whereby the lower the value, the better the performance. MAE was selected as the key criteria to measure average model performance as it has been found to be a more natural measure of average error than RMSE; it is unambiguous [30].

Since no external validation dataset is currently available, model performance was assessed using the internal dataset in two approaches. The combination of model and method with the best goodness-of-fit results in two groups of validation analyses would be the optimal one chosen for the full sample. In the first set of validation analyses (Validation I), the full sample was divided equally into five groups using computer-generated random numbers. Each time, 80% of the sample (i.e. four random groups) was assigned to the “estimation sample” that was used to generate the mapping algorithm, while the remaining 20% of the sample (assigned to the “validation sample”) were used to predict CHU9D utilities based on the above algorithm. This procedure was repeated 5 times, so that each of the five random groups was used in the estimation and validation exercises. Model performance was assessed based on the pooled estimated prediction errors. This validation method is usually referred to as a cross-validation approach in the literature [31,32]. In the second set of validation analyses (Validation II), the mapping algorithms generated through the full sample were tested on three random samples [33]. The three random samples with sample size of 100, 300, and 500 were generated by random selection within the full sample.

## Results

Of the 961 adolescents who consented to take part in the survey, 590 adolescents (61.4%) completed both the CHU9D and KIDSCREEN-10 instruments and had no missing values on age and gender. The mean (standard deviation) CHU9D utility score was 0.808 (0.155) and mean (standard deviation) KIDSCREEN-10 index was 43.737 (7.932). Fifty five percent of respondents were male, the mean (standard deviation) age was 14.5 (2) years, 53% of respondents came from families with high socio-economic status (as defined by the Family Affluence Scale), 92% reported their health status was good, very good or excellent, 11% had a disability. See Table 1 for details.

**Table 1:** **Sample characteristics**

CHU9D utility score, mean (SD)	0.808 (0.155)
KIDSCREEN-10 index, Mean (SD)	43.737 (7.932)
Age (year), Mean (SD)	14.5 (2.0)
Gender, N (%)	
Boys	322 (54.6)
Girls	268 (45.4)
Family affluence scale, N (%)	
High (scores 6–9)	55 (52.7)
Medium (scores 4–5)	223 (37.9)
Low (scores 0–3)	310 (9.4)
Missing	2 (0.3)
Self-reported health, N (%)	
Excellent	145 (24.6)
Very good	268 (45.4)
Good	129 (21.9)
Fair	39 (6.6)
Poor	9 (1.5)
Disability, N (%)	
Yes	67 (11.4)
No	523 (88.6)

CHU9D - Child Health Utility 9D; SD - standard deviation.

Figure 1 shows the kernel density of the CHU9D utility scores and the KIDSCREEN-10 index. The CHU9D utility score is non-normally (left-skewed) distributed while the KIDSCREEN-10 index tends towards a normally distribution (although the null hypothesis for normality was rejected based on Shapiro-Wilk normality test).

Pairwise Pearson’s correlations between each item of the KIDSCREEN-10 index and CHU9D utility score suggest that the strongest correlated item is KS_I1 (“fit and well”, r = 0.488), followed by another 5 items with a correlation higher than 0.4, i.e. KS_I10 (r = 0.447), KS_I3 (r = 0.437), KS_I2 (r = 0.427), KS_I4 (r = 0.416) and KS_I9 (r = 0.406). The remaining 4 items have a correlation with a CHU9D utility score that is lower than 0.4, including KS_I5 (r = 0.365), KS_I8 (r = 0.317), KS_I7 (r = 0.271) and the lowest correlated item was KS_I6 (“been able to do the things that you want to do in your free time”, r = 0.175).

### Prediction of CHU9D utility scores

**Figure 2:**
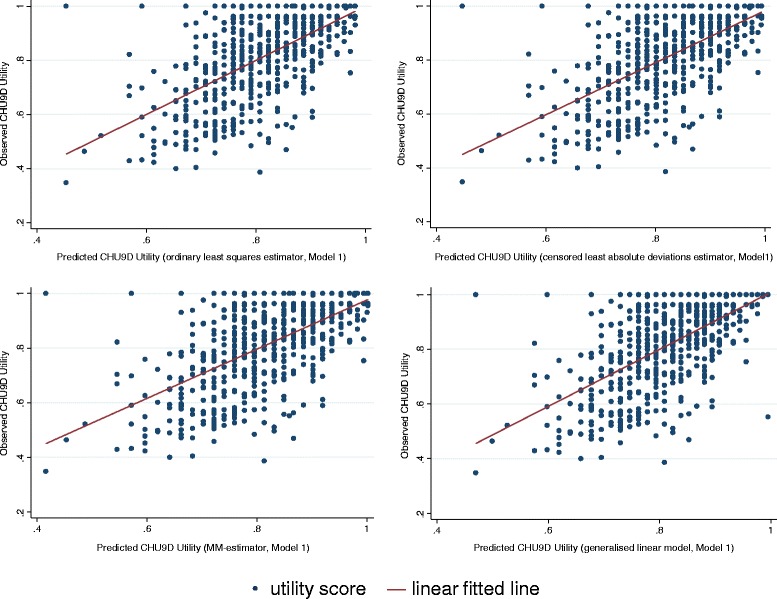
**CHU9D utility scores and the predicted CHU9D utility scores from KIDSCREEN-10 index (Model 1).**

**Figure 3:**
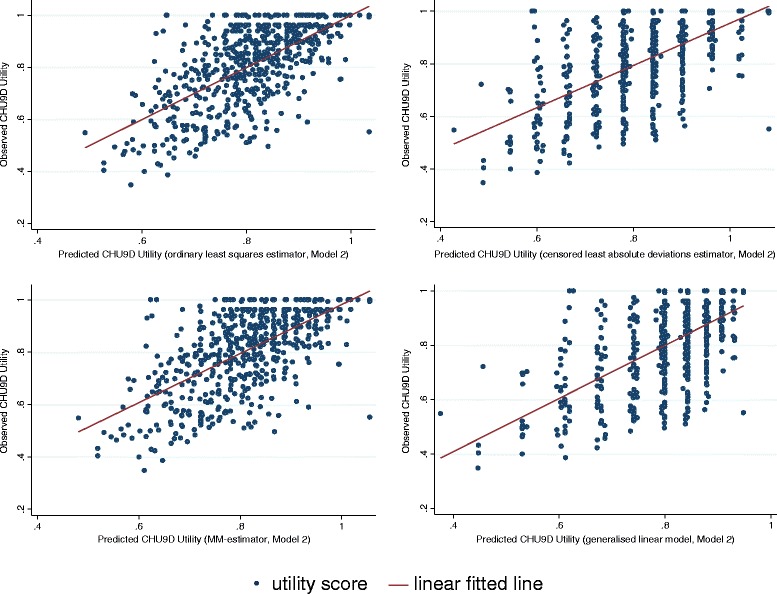
**CHU9D utility scores and the predicted CHU9D utility scores from KIDSCREEN-10 index (Model 2).**

The goodness-of-fit results for different combinations of models and methods of the full sample are reported in Table 2. All estimators tend to over predict the lowest boundary of the utility score and among them, the generalised linear model estimate, based on Model 2, is closest to the observed score (0.3760 vs. 0.3479, Column 2). On the highest boundary of the utility score, estimators may either over or under-estimate the maximum utility. According to the absolute difference, the MM-estimate, based on Model 1, performs the best (1.0019 vs. 1, Column 3). For the two goodness-of-fit indicators, the MM-estimate has the lowest MAE (0.0946, Column 4) and the second lowest RMSE (0.1199, Column 5), whilst the ordinary least squares estimate has the lowest RMSE (0.1193, Column 5) and the second lowest MAE (0.0950, Column 4). Based on the results presented in Table 2, it is reasonable to conclude that the mapping algorithm using the MM-estimator with model 2 specification is preferred based on MAE criteria. Scattergrams of the relationship between the observed and the KIDSCREEN-10 predicted CHU9D utility scores are shown in the Figures 2 and 3.

**Table 2:** **Goodness-of-fit results from full sample**

**Model specification**	**(1)**	**(2)**	**(3)**	**(4)**	**(5)**	**(6)**	**(7)**
	**Mean CHU9D**	**Min CHU9D**	**Max CHU9D**	**MAE**	**RMSE**	**MAE** ^**†**^	**RMSE** ^**†**^
Observed	0.8082	0.3479	1.0000	―	―	―	―
Method 1: Ordinary least squares estimator^‡^							
Model 1	0.8082	0.4535	0.9817	0.0978	0.1238	―	―
Model 2	0.8082	0.4909	1.0342	0.0950^**^	0.1193^*^	0.0946	0.1190
Method 2: Censored least absolute deviations estimator							
Model 1	0.8185	0.4473	0.9944	0.0971	0.1243	―	―
Model 2	0.8179	0.4281	1.0802	0.0971	0.1247	0.0944	0.1219
Method 3: MM-estimator							
Model 1	0.8136	0.4156	1.0019	0.0972	0.1243	0.0971	0.1243
Model 2	0.8146	0.4807	1.0555	0.0946^*^	0.1199^**^	0.0937	0.1193
Method 4: Generalised linear model							
Model 1	0.8082	0.4693	0.9950	0.0975	0.1240	―	―
Model 2	0.8082	0.3760	0.9483	0.0971	0.1217	―	―

CHU9D – Child Health Utility 9D; MAE – mean absolute error; RMSE – root mean squared error.

*denotes the smallest value in the column; **denotes the second smallest value in the column.

^†^The adjusted goodness-of-fit results by specifying the maximum predicted utility score to be 1.

^‡^The R-square statistics for Model 1 and 2 are 0.36 and 0.41, respectively.

### Validation

Table 3 reports two groups of validation analyses results for all combinations of models and methods introduced in the statistical analysis section. According to MAE and RMSE, ordinary least squares and MM-estimates based on the model 2 specification have the best predictive performance across both methods of valuation. Overall the MM-estimates based on the model 2 specification are selected as the preferred model as it performs slightly better using the preferred MAE criteria. The results reported in validation analyses support the conclusion from the full sample analysis that MM-estimator based on Model 2 is the optimal choice if MAE is the key criteria, whilst the ordinary least squares estimator based on Model 2 should be chosen if RMSE is the dominant one.

**Table 3:** **Goodness-of-fit results from validation analysis**

	**Validation I**			**Validation II**								
	**Pooled sample (N = 590)**			**Random sample I (N = 100)**			**Random sample II (N = 300)**			**Random sample III (N = 500)**		
	**Mean utility**	**MAE**	**RMSE**	**Mean utility**	**MAE**	**RMSE**	**Mean utility**	**MAE**	**RMSE**	**Mean utility**	**MAE**	**RMSE**
	**(1)**	**(2)**	**(3)**	**(4)**	**(5)**	**(6)**	**(7)**	**(8)**	**(9)**	**(10)**	**(11)**	**(12)**
Observed	0.8082	―	―	0.8265	―	―	0.8094	―	―	0.8102	―	―
Method 1: Ordinary least squares estimator												
Model 1	0.8085	0.0982	0.1245	0.8166	0.0874	0.1091	0.8107	0.0938	0.1205	0.8111	0.0985	0.1248
Model 2	0.8088	0.0963^**^	0.1209^*^	0.8127	0.0845^**^	0.1054^**^	0.8112	0.0943	0.1187^*^	0.8104	0.0947^**^	0.1194^*^
Method 2: Censored least absolute deviations estimator												
Model 1	0.8202	0.0993	0.1268	0.8274	0.0867	0.1084	0.8211	0.0931^*^	0.1209	0.8214	0.0977	0.1253
Model 2	0.8207	0.1006	0.1273	0.8378	0.0866	0.1097	0.8358	0.0946	0.1227	0.8344	0.0954	0.1232
Method 3: MM-estimator												
Model 1	0.8133	0.0983	0.1253	0.8232	0.0865	0.1082	0.8164	0.0931^*^	0.1208	0.8167	0.0977	0.1253
Model 2	0.8147	0.0962^*^	0.1216^**^	0.8201	0.0842^*^	0.1053^*^	0.8181	0.0937^**^	0.1193^**^	0.8168	0.0944^*^	0.1200^**^
Method 4: Generalised linear model												
Model 1	0.8082	0.0977	0.1243	0.8149	0.0881	0.1097	0.8104	0.0940	0.1215	0.8108	0.0984	0.1252
Model 2	0.8085	0.0979	0.1226	0.8104	0.0920	0.1144	0.8085	0.0964	0.1206	0.8092	0.0967	0.1211

MAE – mean absolute error; RMSE – root mean squared error.

*denotes the smallest value in the column; **denotes the second smallest value in the column.

### Mapping equations

The detailed regression results using the full sample are reported in Table 4. Gender was consistently insignificant in all scenarios. Age was found to be significant only one occasion where the ordinary least squares estimator was applied. For all other three estimators, age was insignificant. Considering these findings, both gender and age were not included in the final regression equations. For Model 1, both the original KIDSCREEN-10 index and its squared term were found to be robustly significant (P < 0.05) in three estimates (ordinary least squares, censored least absolute deviations and MM-estimator), indicating the existence of the non-linear relationship between the two instruments. The generalised linear model incorporates the nonlinear relationship between dependent and independent variables through the link function, and as shown in Model 1, the coefficient of the KIDSCREEN-10 index was statistically significant (P < 0.05) whilst the squared term was insignificant and not included.

**Table 4:** **Mapping equations from KIDSCREEN-10 index to Child Health Utility 9D utility scores**

	**Ordinary least squares estimator**		**Censored least absolute deviations estimator**		**MM-estimator**		**Generalised linear model**	
	**Coeff.**	**SE** ^**†**^	**Coeff.**	**SE** ^**†**^	**Coeff.**	**SE** ^**†**^	**Coeff.**	**SE** ^**†**^
*Model 1*								
KS	0.043515	0.005291*	0.046580	0.006828*	0.049504	0.006682*	0.092650	0.008747*
KS^2^	−0.000334	0.000053*	−0.000359	0.000072*	−0.000384	0.000070*		
Constant	−0.435412	0.129225*	−0.510120	0.160989*	−0.593052	0.157245*	−2.472760	0.359525*
*Model 2*								
KS_I1	0.035797	0.008005*	0.059820	0.009940*	0.037867	0.010995*	0.296834	0.042888*
KS_I2	0.017943	0.007725*			0.023085	0.009292*		
KS_I3	0.037163	0.008005*	0.039315	0.011111*	0.037192	0.009329*	0.331778	0.040524*
KS_I4	0.022713	0.006543*	0.027291	0.010421*	0.021284	0.007952*		
KS_I9	0.016046	0.007037*			0.024877	0.008434*		
KS_I10	0.027138	0.008991*	0.060152	0.010321*	0.022256	0.010361*	0.300356	0.041449*
Constant	0.250215	0.029866*	0.156848	0.053203*	0.222655	0.034914*	−1.735730	0.167557*

^†^Heteroskedasticity robust standard errors (SE). *significant at 5%. For generalised linear model, *binomial* family and *logit* link were used.

KS – the KIDSCREEN-10 index; KS_I1 – “fit and well”, KS_I2 – “energy”, KS_I3 – “sad”, KS_I4 – “lonely”, KS_I9 – “got on well at school”, KS_I10 – “been able to pay attention”.

In Model 2, the stepwise selected significant KIDSCREEN-10 items are the key predictors. As can be seen, not all of the 10 items were significant, but for all statistically significant items the positive coefficients were consistent with the expectation that a high item score (better health) is associated with a higher utility. The potential multicollinearity issue was detected using variance inflation factor and the mean/highest variance inflation factor in this case is 1.88/2.01, suggestion that none of the items suffered from multicollinearity and can be included simultaneously in the regressions. The items that were found to be robustly non-significant across four estimators were KS_I5 (“had enough time for yourself”), KS_I6 (“been able to do the things that you want to do in your free time”), KS_I7 (“parent(s) treated you fairly”) and KS_I8 (“had fun with friends”). This is consistent with the findings from the pairwise correlation analysis, specifically that all four items exhibited a relative lower correlation relationship with CHU9D (r < 0.4). A bootstrap stepwise ordinary least squares regression technique (with 100 replications) was also conducted. Ranked by the number of times each variable is selected, KS_I3 topped the list (100 out of 100 times been selected), followed by KS_I1 (99 out of 100), KS_I10 (93 out of 100), KS_I4 (91 out of 100), KS_I9 (59 out of 100), KS_I2 (55 out of 100), KS_I7 (36 out of 100), KS_I8 (29 out of 100), KS_I5 (21 out of 100), and KS_I6 (8 out of 100). Consistent with these findings, KS_I7, KS_I8, KS_I5, and KS_I6 demonstrate the least importance in mapping onto the CHU9D utility. See Table 4 for the detailed regression outputs of four estimators. Based on the MAE result discussed above, the optimal equation used to predict the CHU9D utility based on KIDSCREEN-10 items would be:

CHU9D utility score = 0.222655 + 0.037867*KS_I1 + 0.023085*KS_I2 + 0.037192*KS_I3 + 0.021284*KS_I4 + 0.024877*KS_I9 + 0.022256*KS_I10.

As previously highlighted, there are currently two preference based scoring algorithms available for the CHU9D, the original one generated by the standard gamble method with the UK adult general population and a newly developed one generated by the best-worst scaling method with the Australian adolescent general population and anchored on the 1–0 full health-dead scale using the UK values. The utility scores generated by application of the two scoring algorithms are highly correlated (r = 0.97). The correlation between each item of the KIDSCREEN-10 instrument and each of the two utility scores are almost identical. Owing to word limits, the analyses presented here were based upon the Australian adolescent general population scoring algorithm. The key mapping equations (corresponding to those reported in Table 4) from the KIDSCREEN-10 index to the CHU9D utility scores based upon the UK adult scoring algorithm are also reported in the Table 5 for the readers’ interest. The goodness-of-fit results also suggest that the ordinary least squares and MM-estimates based on the Model 2 specification had the best predictive performance, and the MM-estimates based on the Model 2 specification is selected as the preferred model using MAE.

**Table 5:** **Mapping equations from KIDSCREEN-10 index to UK Child Health Utility 9D utility scores**

	**Ordinary least squares estimator**		**Censored least absolute deviations estimator**		**MM-estimator**		**Generalised linear model**	
	**Coeff.**	**SE** ^**†**^	**Coeff.**	**SE** ^**†**^	**Coeff.**	**SE** ^**†**^	**Coeff.**	**SE** ^**†**^
*Model 1*								
KS	0.032434	0.004171*	0.029623	0.004257*	0.032500	0.004142*	0.082786	0.007694*
KS^2^	−0.000249	0.000041*	−0.000218	0.000045*	−0.000246	0.000041*		
Constant	−0.075688	0.102939	−0.006549	0.097355	−0.077689	0.102295	−1.749110	0.316079*
*Model 2*								
KS_I1	0.026771	0.006051*	0.025167	0.008471*	0.022931	0.007461*	0.257334	0.038624*
KS_I2	0.010975	0.005552*	0.022333	0.006579*	0.018505	0.006152*		
KS_I3	0.029050	0.006725*	0.028383	0.007295*	0.022030	0.006130*	0.298660	0.038848*
KS_I4	0.015820	0.005122*	0.015550	0.006502*	0.014684	0.005477*		
KS_I9	0.013502	0.005639*	0.025567	0.004682*	0.023993	0.004852*		
KS_I10	0.020093	0.007056*			0.012365	0.006212*	0.271802	0.037553*
Constant	0.437368	0.024759*	0.440167	0.032133*	0.451961	0.024881*	−1.080010	0.162497*

Note: Predicted utility values are for the UK scoring algorithm of the Child Health Utility 9D based on adult values elicited using standard gamble.

^†^Heteroskedasticity robust standard errors. *significant at 5%. For generalised linear model, *binomial* family and *logit* link were used.

KS – the KIDSCREEN-10 index; KS_I1 – “fit and well”, KS_I2 – “energy”, KS_I3 – “sad”, KS_I4 – “lonely”, KS_I9 – “got on well at school”, KS_I10 – “been able to pay attention”.

## Discussion

The measurement and valuation of the HRQoL of children and adolescents is increasingly being recognised as an important component of economic evaluations of health care treatment and preventive programs targeted for young people. The KIDSCREEN-10 instrument has been validated across several European countries for the measurement of health status and since its development in 2004 the instrument has been also widely used across countries. However, a current limitation of the KIDSCREEN-10 is the absence of preference weights meaning that the measure cannot be used directly to estimate QALYs for use in cost-utility analyses. This study has developed a mapping algorithm that can be used to predict CHU9D utility scores based on the KIDSCREEN-10 index. The utilisation of the algorithm will enable cost-utility analyses to be conducted within studies where health outcomes were assessed using only the KIDSCREEN-10 index.

There are two main strengths of this study. Firstly, the target and base measures are both generic HRQoL instruments and as such they have a conceptual overlap between each other. This is an important determinant to the success of mapping analysis [9,22,34]. Secondly, multiple estimators that are appropriate for the data have been adopted to explore the optimal mapping algorithms [22]. Specifically, we have used the MM-estimator, an effective robust estimator to map the KIDSCREEN-10 to CHU9D. The MM-estimator has not to our knowledge been previously used in mapping and in this dataset outperforms the censored least absolute deviations and generalised linear model techniques that have been used previously in the mapping literature, and performs similarly to ordinary least squares in this dataset. As the MM-estimator offers some theoretical advantages over ordinary least squares estimator and performs similarly for this reason it is our preferred model here. The model performance as indicated by MAE (0.0946) of the preferred MM-estimate model based on the Model 2 specification is within the range reported by previously published studies (0.0011 to 0.19) [9].

Despite our preference for the MM-estimator, it should be noted that these two estimators do perform similarly. In terms of their predictive ability as the RMSE value (0.1193) of the optimal ordinary least squares estimate is also within the published ranges (0.084 to 0.2) [9]. The largely comparable predictive performance of ordinary least squares and MM-estimator models, despite the MM-estimator overcoming the theoretical limitations of ordinary least squares estimator for the analysis of CHU9D, is of interest. However in the literature this has also been found in some studies mapping onto the EQ-5D using ordinary least squares estimator and other models overcoming the theoretical limitations of ordinary least squares estimator [22].

Although aggregated sample/group level and dis-aggregated individual level predictions of CHU9D utility scores can be incorporated within economic evaluation, it is recommended that only the aggregated sample/group level prediction be adopted based on the current algorithm. At the individual level the predicted utility scores are less reliable as the prediction error could be large as indicated in the Figures 2 and 3. The over-prediction at the lower end of utility values is an issue that not uncommon in the mapping analysis where regression technique is used [35]. Furthermore, as can be seen from Columns (2) and (3) of Table 2, there is no guarantee that the predicted utility will lie within the observed ranges if the transformation algorithm is based upon ordinary least squares estimator, censored least absolute deviations or MM- estimators. Some studies have suggested that in practice if the predicted utility fell outside the defined range, then it should be truncated to the appropriate boundary value (e.g. Sullivan and Ghushchyan [29], Wu *et al*. [31], Payakachat *et al*. [36]). Following this suggestion, the predicted utility score should be specified to 1 if the prediction is larger than 1. How this modification will change the goodness-of-fit results in our sample is shown in Columns (6) and (7) of Table 2. As can be seen, this adjustment always improves the goodness-of-fit results.

This study has some limitations. Response rates and data quality are two potential issues with online modes of survey administration. On-line modes of administration are increasingly familiar, particularly for young people and have the potential to engage large numbers of community based adolescents who would otherwise be more difficult to reach. It is possible to include checks for data quality in on-line surveys and we have taken care to scrutinise the data generated for illogical responses and to check that respondents appeared to understand the task adequately. It is also important to note that other modes of survey administration including self-completion questionnaires and interviews may also be plagued by low response rates and issues of data quality.

In relation to the modelling approach adopted it is important to highlight that model performance was validated using the internal dataset only. A cross-validation would be ideal once a suitable external dataset becomes available. In addition, the study sample was relatively healthy and as such it is also possible that the best performing model specification and type would have differed if the mapping algorithms had been estimated using a dataset with a larger number of respondents in poorer health. Therefore, an external validation using a patient sample is recommended prior to using these mapping algorithms in a dataset with children in poor health. An alternative mapping method, the linking approach that has not yet been empirically tested could be explored in future studies [37].

## Conclusion

When a preference based instrument has not been included in a study to enable QALYs to be estimated for use in cost-utility analyses, the adoption of a mapping approach from a non-preference based instrument to obtain health state utilities served as a second best alternative facilitating cost-utility analyses. This paper has produced a mapping algorithm to generate a CHU9D utility score from KIDSCREEN-10 items. The preferred model is the MM-estimate with stepwise selected KIDSCREEN-10 item scores as the predictors (i.e. Model 2 in Table 4) according to the MAE. The ordinary least squares estimate with stepwise selected KIDSCREEN-10 item scores as the predictors also show good performance based on RMSE.
